# Comparison Between Levothyroxine and Lifestyle Intervention on Subclinical Hypothyroidism in Women: A Review

**DOI:** 10.7759/cureus.38309

**Published:** 2023-04-29

**Authors:** Claire L Matlock, Anna R Vanhoof, Shahid B Rangrej, Rajni Rathore

**Affiliations:** 1 Medical School, Saint James School of Medicine, Arnos Vale, VCT; 2 Anatomy/Research, Saint James School of Medicine, Arnos Vale, VCT; 3 Medical Education, Saint James School of Medicine, Arnos Vale, VCT

**Keywords:** exercise, iodine, diet, lifestyle intervention, women, thyroid, subclinical hypothyroidism

## Abstract

Subclinical hypothyroidism (SCH) or “mild thyroid failure” is defined as elevated serum thyroid-stimulating hormone (TSH) in the presence of normal free thyroxine (T4). The incidence of SCH is estimated at 4.4-8.5% of the general population and occurs more frequently in women. Given that it falls below the diagnostic threshold, SCH is monitored rather than treated. Its management is a common topic of debate as SCH frequently progresses into overt hypothyroidism and is linked to long-term hyperlipidemia, endothelial dysfunction, cardiovascular disease, heart failure, and cerebrovascular disease. Premature hormone administration and lifestyle interventions have been explored as treatment options to mitigate the symptoms of SCH. Our review compares both modalities’ efficacy and potential for standardized clinical practice. A trial of levothyroxine demonstrated significant results in specific SCH demographics, such as patients who are pregnant or trying to conceive, those with goiter, those with thyroid peroxidase (TPO) antibody status, those with steadily increasing TSH, children, and adolescents. All other SCH patients presenting with chronic symptoms may also be reasonably considered for a three- to six-month trial of treatment. Lifestyle modifications through improved sleep hygiene, a diet within the recommended daily allowance (RDA) for iodine and selenium, increased exercise, and smoking cessation also proved efficacious. Our findings indicate that a synergistic approach to treatment is most favorable. Lifestyle modifications neither show adverse effects nor contraindications and can be safely recommended alone or alongside levothyroxine for the treatment of SCH.

## Introduction and background

The thyroid gland regulates metabolic rate and growth. A deficiency in its hormone production yields a broad constellation of symptoms. In women, this presents clinically as fatigue, weight gain, cold extremities, myasthenia, dry skin, constipation, hyperlipidemia, depression, poor memory, voice changes, menstrual abnormalities, and miscarriage, amongst other symptoms [1,2]. Subclinical hypothyroidism (SCH) is defined as mildly elevated thyroid-stimulating hormone (TSH) above the normal limit (0.4-4.5 mIU/L) and below that of overt hypothyroidism (<10.0 mIU/L) despite normal serum levels of free thyroxine (T4) [3]. SCH can thus be understood as the ambiguous diagnostic range between healthy and sick. It is a common occurrence with an estimated prevalence of 7.5-8.5% in women and 4.4% in men, with no previous thyroid-related issues [1]. Among its many symptoms, the chief complaints of SCH patients presenting to their physicians are fatigue and weight gain [4]. Its prevalence increases with age and remains more common in females than males until approximately the sixth decade of life [3].

The clinical significance surrounding therapy for SCH is a common topic of debate given that it frequently progresses into overt hypothyroidism and is linked to long-term hyperlipidemia, endothelial dysfunction, cardiovascular disease, heart failure, and cerebrovascular disease [1,3,5,6]. Levothyroxine is widely accepted as the first-line treatment for overt hypothyroidism. Questions have been raised regarding whether the upper limit required for diagnosis could itself be the issue [7-9]. Lowering the cutoff value would transition more SCH patients into the overt hypothyroid category, qualifying them for hormone therapy. However, this would create an estimated four-fold increase - an additional 22-28 million Americans - in overt diagnoses and an accompanying burden on the healthcare system [3].

Many studies have assessed the effectiveness of premature hormone administration for SCH patients. Likewise, lifestyle interventions, such as diet and exercise, have been implemented as strategies to mitigate symptoms. However, there is a lack of current research comparing the two modalities’ efficacy and potential for standardized clinical practice. Our review of the literature bridges the gap between these two treatments. We analyzed and consolidated the literature to evaluate levothyroxine versus lifestyle intervention as clinical strategies in the management of SCH.

## Review

Methodology

In March 2022, a review was conducted using electronic databases including PubMed, MEDLINE and the Saint James School of Medicine Library. The keywords “subclinical hypothyroidism,” “women,” “levothyroxine,” “lifestyle intervention,” “diet,” and “exercise” with the use of Boolean operators, “AND,” “OR” and “NOT,” were used to identify relevant studies comparing treatment options for SCH.

The following inclusion criteria were applied: (1) A scholarly and/or peer-reviewed source; (2) research conducted within the past 20 years, beginning at the date of research; (3) articles published in the English language; (4) dietary intake measured through a standardized criterion, such as the Food Frequency Questionnaire (FFQ), caloric intake, nutrient intake and/or food groups, dietary patterns, length of time adhering to the diet, etc.; (5) exercise and physical activity measured through a standardized criterion, such as type of exercise, length of activity, total caloric energy expenditure, length of time adhered to an exercise program, etc.

Using the above-defined criteria, our search yielded 183 full-text articles which were further assessed by authors. Articles were evaluated for comparisons between levothyroxine treatment and other lifestyle interventions in women. For consistency, all studies selected defined SCH as a TSH level between 4 and 10 mU/I. Of these, 27 studies were eligible: 15 systematic reviews, three meta-analyses, three cross-sectional studies, two randomized control trials, two cohort studies, one crossover study, and one case report. Our results are presented in a Preferred Reporting Items for Systematic Reviews and Meta-Analyses (PRISMA) diagram. 

**Figure 1:**
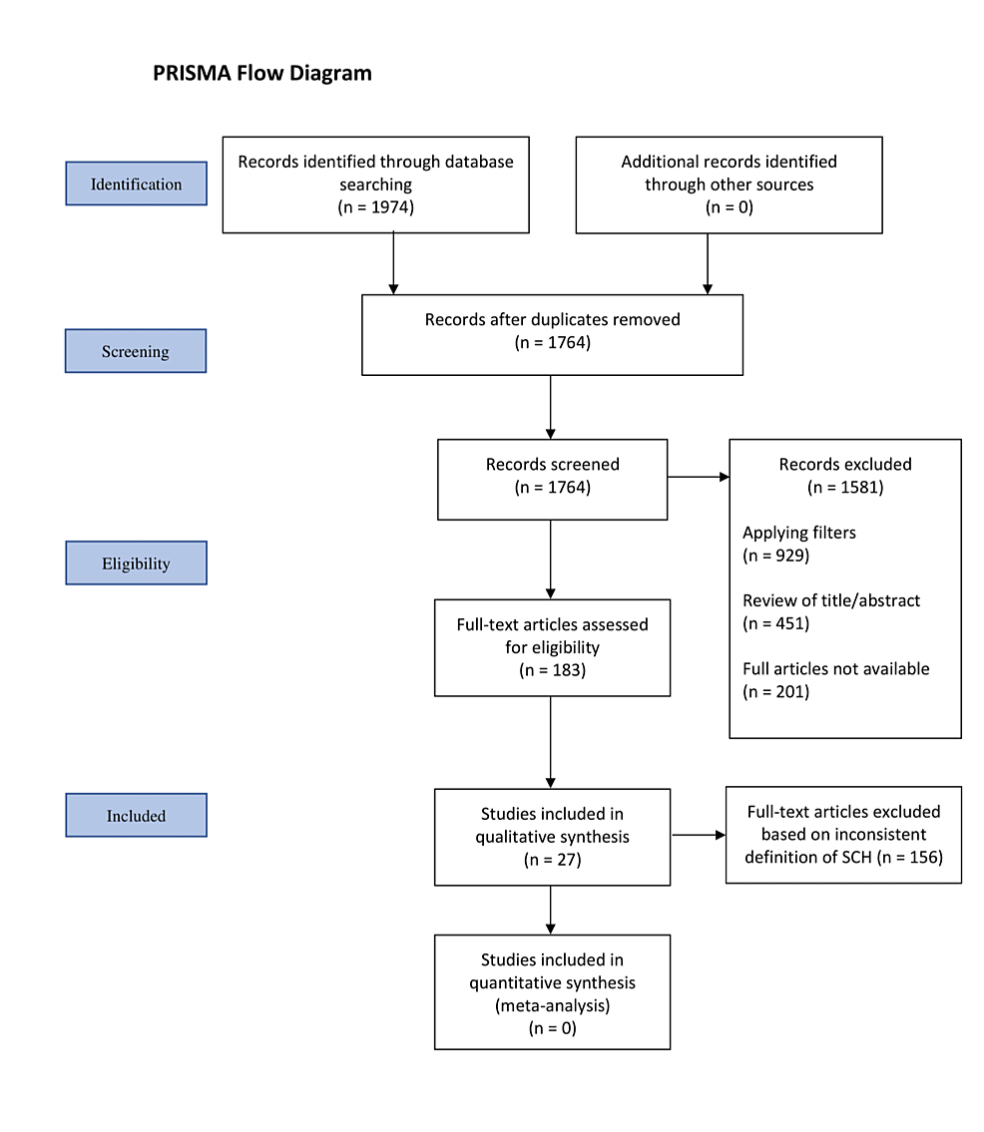
PRISMA Flow Diagram SCH: Subclinical hypothyroidism

Modifying the upper limit

Prior to considering treatment for SCH, many studies question if the diagnostic threshold itself might be an issue [7-9]. Currently, patients presenting with TSH > 10.0 mIU/L qualify for levothyroxine therapy. This is consistently acknowledged across the literature and implemented as standardized practice in outpatient clinics. Anyone below this cutoff is not routinely treated. One study demonstrated that of 12,667 SCH patients left untreated, 62% measured TSH levels within the normal reference range when retested five years later [10]. Another study yielded similar results, showing normalized TSH levels amongst 52% of middle-aged SCH patients after a mean follow-up of 32 months [11]. Consequently, the same study found that only 26.8% of patients went on to develop overt hypothyroidism. However, this number was as low as 2.9% in one five-year cohort study [10]. Additionally, it should be considered that a mildly elevated TSH does not necessarily indicate thyroid failure. Secondary causes of increased TSH are numerous and often transient: post-viral thyroiditis, postpartum thyroiditis, granulomatous thyroiditis, thyroid hormone resistance, prior radiation therapy to the neck region, variability in the assay or contamination with interfering heterophile antibodies, etc. [3]. This begs the question if SCH is better left untreated. Yet, of the estimated millions of Americans with SCH, many thousands present to their doctors with chronic symptoms only to encounter no standardized options for care. 

Treating SCH patients yielded mixed results. A double-blind crossover 12-month study of thyroxine treatment for women with SCH, found that only four of the 17 women who completed the study showed improvement after six months [12]. Researchers concluded that only one in four women with SCH will likely benefit from levothyroxine treatment. Another study, conducting a six-month randomized trial of thyroxine treatment amongst 40 SCH women, noted more adverse effects when treated with levothyroxine than positive outcomes [4]. They found no significant changes in baseline metabolic, lipid or anthropometric levels six months post-levothyroxine treatment in either the control or placebo group. Results did, however, show a significant increase in anxiety scores amongst the thyroxine group as compared to the placebo group.

Conversely, one observational study showed great benefit in treating patients (<70 years) to reduce SCH sequelae, namely coronary heart disease [6]. Other data reinforce this, explaining that levothyroxine leads to improved lipid profiles, endothelial function, and echocardiographic features, but not a reduction of the cardiovascular disease itself [13,14]. Yet, it is also said that serum TSH level rises progressively with age and thus SCH diagnoses might be overestimated in older populations [15]. Given the variability of these results, altering the diagnostic criteria of hypothyroidism via lowering the TSH cutoff score, does not appear to be an effective solution at this time.

Who should be treated

Treating with levothyroxine, on a case-by-case basis, appears to be a more strategic approach. Evidence is statistically significant across eight of the analyzed studies supporting treatment on an individualized basis. There is a nearly unanimous agreement for treating pregnant SCH women, those suffering from infertility and/or wishing to conceive [1,16-19]. Maternal thyroid hormones play an important role in fetal development, especially neuropsychiatric. Deficiency carries the risk of adverse obstetric outcomes. For this reason, overtly hypothyroid mothers are serially monitored throughout pregnancy and receive a step-up dose of levothyroxine. It is recommended that pregnant SCH patients be treated similarly by having low thyroid levels amended as soon as possible [1,3]. Physicians are encouraged to start patients on a full replacement dose of levothyroxine as soon an increase in TSH is observed [1].

Beyond this demographic, other SCH groups appear to benefit from premature hormone administration. Those with goiter, progressively increasing TSH levels, and elevated thyroid peroxidase (TPO) antibodies are at greater risk for progression into over-hypothyroidism and should be considered for treatment [3,17]. Children and adolescents, given the importance of thyroxine on development, can also benefit from treatment [3,20]. Lastly, all other patients who do not meet these criteria and yet present with chronic symptoms might be considered by their physician for treatment. That is, anyone whose TSH score is within the range of 4-10mU/I, through patient preference and physician accordance, may trial a three to six month period of levothyroxine [21]. If symptoms resolve, patients can be continued on levothyroxine. If symptoms do not resolve, doctors are encouraged to consider an alternative diagnosis and check for TPO antibody status.

Evidently, the decision to initiate treatment of SCH should follow an individualized approach. Physicians are encouraged to weigh the risks and benefits of starting their patients on levothyroxine. Details regarding how to assess those advantages and disadvantages were outlined in one study [5]. Researchers stated that physicians should consider a patient’s age, symptoms, thyroid antibodies, and risk for comorbidities when deciding whether to pharmacologically treat SCH.

Lifestyle interventions 

Non-pharmaceutical options to treat SCH have also been trialed, including diet, exercise, and lifestyle habits. Some studies prove it possible to lower TSH scores back to euthyroid status, while others simply reduce undesirable symptoms. There is a strong association between sleep, smoking, diet, and exercise when it comes to thyroid function [1,22]. Poor sleep quality was proven to carry an increased risk for SCH [21]. Likewise, a diet too low or too high in iodine was found to increase one’s risk [22-24]. It is important to consider that the characteristic symptoms of hypothyroidism, such as fatigue, weight gain and depression, make it less likely that patients will engage in certain beneficial lifestyle habits such as regular exercise. One cross-sectional study measuring physical activity in women found that the duration of physical activity, number of daily steps and muscle strength was lower in the group of women with SCH than in their euthyroid counterparts [25]. It can be understood that sedentary lifestyles are associated with higher TSH scores and yet higher TSH scores exacerbate sedentary lifestyles. Decreased exercise tolerance is therefore a relevant obstacle to implementing well-intentioned medical advice.

Diet, therefore, seems to be a more realistic place to make initial changes. Although not conducted amongst women, a randomized control trial of SCH in 62 children saw that six months of eating certain foods, including green vegetables, beef, whole milk, and butter, with no other aspects of diet changed, significantly improved their chief complaint of tiredness when compared to the control group [20]. These dietary modifications did not unfavorably augment the intervention group’s lipid profile or BMI. One European study found that selenium supplementation maintained or lowered TSH levels. It also reduced thyroid antibodies, T4/T3 ratio, and overall oxidative stress and inflammation [26].

Iodine, however, is by far the most studied mineral with regard to thyroid function. There was substantial evidence in three studies to suggest that either a deficiency or excess of iodine resulted in a higher risk for SCH [22-24]. A cross-sectional study analyzing the effect of lifestyle on thyroid function found that iodine imbalance played a significant role in thyroid secretory capacity and its deficiency or excess carried an increased risk of SCH [23]. Investigating two Chinese communities with different iodine intake levels, researchers found that those living in the community with a higher iodine intake were at significantly higher risk for developing SCH than their counterparts in the community with a lower iodine intake. Researchers inferred that excess iodine intake was a precursor for SCH and subsequent overt hypothyroidism. This was supported by a large-scale cohort study in which SCH patients, in an iodine-replete area, were restricted back to the recommended daily allowance (RDA) level of 150 μg/day and were subsequently able to decrease and, in some cases, entirely normalize their TSH levels [24]. In comparison to the two control groups who were not iodine-restricted, the experimental group was able to significantly improve TSH levels and increase T4 levels. They concluded that restriction of excess iodine intake could be a primary treatment option for SCH patients.

Conversely, supplementing iodine in SCH patients whose diets are intrinsically low in the mineral yields promising results. One case report showed significant SCH improvement after supplementing iodine when a patient’s diet was originally low [27]. In this report, a postmenopausal woman with SCH, dyslipidemia, and obesity was monitored for one month while eating the RDA of iodine. She received guidance on which foods are rich in minerals and how to practically incorporate them into her diet. The results were extremely promising. Not only did she improve her TSH level, but her total cholesterol decreased and her body mass index fell (30.13 to 28.5 kg/m2). These results were energizing to the patient. It can be reasonably inferred that her improvement would make it easier and more enjoyable to engage in other beneficial lifestyle habits, such as increased physical activity. Overall, it is evident that lifestyle modifications impact thyroid function and carry the power to improve SCH symptoms; however, more targeted studies would help substantiate this claim. 

Dual approach

There is evidence that SCH can be improved in women with and without medication. Given that cessation of smoking and improvements in quality of sleep, diet, and exercise carry no side effects, but rather only serve to reduce all-cause mortality, recommending them as a treatment option is safe and sensible. Additionally, implementing levothyroxine on a case-by-case basis, with frequent monitoring, is a reasonable approach to pharmaceutical treatment. While our review originally sought to compare both modalities and find the superior option, the results speak to a more synergistic, individualized approach. Certain demographics appear to benefit from levothyroxine while others do not. The research data on lifestyle interventions is limited and yet promising. Either treatment option, or both combined, offer a viable solution for many millions of Americans who fall below the clinical threshold and yet deserve to feel healthy. The results obtained throughout our analysis are presented in Table 1.

**Table 1:** Characteristics of the studies in this literature review

Authors (Year)	Study Type	Country	Participants and Sample Size	Findings
Pharmaceutical Intervention				
Díez et al. [11]	Cohort	Spain	107 adults >55 years (93 women, 14 men)	After a mean follow-up of 32 months, TSH levels normalized amongst 52% of SCH patients. 26.8% developed overt hypothyroidism.
Kong et al. [4]	Randomized Control Trial	United Kingdom	40 women	A 6-month randomized trial of thyroxine treatment (n=23) versus placebo (n=17) amongst 40 SCH women found no clinically relevant benefits in the treatment group. However, anxiety scores increased as an adverse outcome in the treatment group.
Meyerovitch et al. [10]	Meta-analysis	Israel	422,242 patients (95% euthyroid, 1.2% hyperthyroid, 3.0% SCH, 0.7% overt hypothyroidism)	Of SCH patients left untreated, 62% measured TSH levels within the normal reference range when retested 5 years later
Nyström et al. [12]	Crossover	Sweden	12 women	A double-blind crossover 2x6 months study of thyroxine treatment in 17 women with SCH found that only 4 of the 17 women who completed the study showed improvement after 6 months. Study concluded that only 1 in 4 SCH women benefit from treatment.
Rodondi et al. [6]	Meta-analysis	Switzerland	55,287 adults (6.2% SCH, 93.8% euthyroid)	Higher TSH scores were associated with an increased risk of CHD (coronary heart disease) events and morality. Risk increased with heightened TSH score. There was proven benefit in treating SCH patients (<70 years) to reduce CHD
Lifestyle Intervention				
Joung et al. [24]	Cohort	Korea	126 adults	SCH patients, in an iodine-replete area, were either restricted back to RDA levels (n=62) or ate normally and did not restrict iodine-rich foods (n=64). Those who restricted iodine were able to decrease and, in some cases, entirely normalize their TSH levels.
Tanriverdi et al. [25]	Cross-sectional	Turkey	60 women	SCH women (n=32) had lower physical activity levels, including exercise duration, number of steps, handgrip, and quadriceps muscle strength, than their healthy counterparts (n=28).
Teng et al. [23]	Cross-sectional	China	3813 adults	Amongst 2 Chinese communities, a higher iodine intake community was correlated with a significantly higher risk for developing SCH than the control lower iodine intake community. Excess iodine was concluded to be a precursor for SCH and subsequent overt hypothyroidism.
van der Gaag et al. [20]	Randomized control trial	Netherlands	62 children (1-12 years)	SCH children were monitored for 6 months eating either a standard control diet (n=32) or an experimental diet (n=29). The experimental diet included certain foods such as: green vegetables, beef, whole milk, and butter. Neither group improved their TSH score. However, the experimental group significantly improved their chief complaint of tiredness.
Wu et. al. [22]	Cross-sectional	China	318 adults (SCH n=159, [males=81, females=78]; euthyroid n=159, [males=87, females=72])	Poor overall sleep quality as well as dietary iodine excess was found to increase the risk of SCH. In the SCH group, duration of physical activity, number of daily steps, and muscle strength was lower in those with SCH versus their euthyroid counterparts.

## Conclusions

SCH is a growing public health concern as its prevalence within the American population continues to increase. Altering the diagnosis threshold, so as to qualify more subclinical patients for treatment, does not appear realistic at this time. However, treating patients on an individualized basis demonstrates promising results. Both strategies - pharmaceutical intervention and lifestyle modifications - prove statistically significant in their ability to reduce patient TSH levels and mitigate undesirable symptoms. There is nearly unanimous agreement to treat SCH women who are pregnant or planning to conceive. There should be a low threshold for initiating hormone therapy in this critical group. Additionally, those with goiter, TPO antibody status, steadily increasing TSH levels, children, teenagers, and anyone else with unrelenting symptoms can be considered for treatment. A three to six month trial of levothyroxine with follow-up monitoring is considered reasonable. Lifestyle interventions also proved efficacious in the significant reduction of symptoms. Sleep hygiene, smoking cessation, increased exercise, and dietary selenium all contribute to improved TSH levels. Most notably, dietary iodine is tightly correlated with thyroid function. Restricting or repleting patient levels back to iodine's RDA proved efficacious in treating SCH. Research has shown the effectiveness of both modalities and favors a synergistic approach to treatment. Those suffering from symptoms can benefit from a trial of levothyroxine and can also make lifestyle modifications to assist in the improvement and/or resolution of SCH.
