# Correction: Comparison Between Levothyroxine and Lifestyle Intervention on Subclinical Hypothyroidism in Women: A Review

**DOI:** 10.7759/cureus.c114

**Published:** 2023-05-05

**Authors:** Claire L Matlock, Anna R Vanhoof, Shahid B Rangrej, Rajni Rathore

**Affiliations:** 1 Medical School, Saint James School of Medicine, Arnos Vale, VCT; 2 Anatomy/Research, Saint James School of Medicine, Arnos Vale, VCT; 3 Medical Education, Saint James School of Medicine, Arnos Vale, VCT

This article has been corrected at the request of the submitting author to change affiliation information.

For each of the four authors, their affiliation information has been changed from "Saint James School of Medicine, Chicago, USA" to "Saint James School of Medicine, Arnos Vale, VCT".

The authors deeply regret that this error was not identified and addressed prior to publication.

 

